# Psychosocial and biological risk factors of anxiety disorders in adolescents: a TRAILS report

**DOI:** 10.1007/s00787-020-01669-3

**Published:** 2020-10-28

**Authors:** Altanzul Narmandakh, Annelieke M. Roest, Peter de Jonge, Albertine J. Oldehinkel

**Affiliations:** 1 Department of Psychiatry, University Medical Center of Groningen, University of Groningen, Groningen, The Netherlands; 2 Department of Developmental Psychology, University of Groningen, Groningen, The Netherlands

**Keywords:** Anxiety disorder, Psychobiological risk factors, Adolescence

## Abstract

Anxiety disorders are a common problem in adolescent mental health. Previous studies have investigated only a limited number of risk factors for the development of anxiety disorders concurrently. By investigating multiple factors simultaneously, a more complete understanding of the etiology of anxiety disorders can be reached. Therefore, we assessed preadolescent socio-demographic, familial, psychosocial, and biological factors and their association with the onset of anxiety disorders in adolescence. This study was conducted among 1584 Dutch participants of the TRacking Adolescents’ Individual Lives Survey (TRAILS). Potential risk factors were assessed at baseline (age 10–12), and included socio-demographic (sex, socioeconomic status), familial (parental anxiety and depression), psychosocial (childhood adversity, temperament), and biological (body mass index, heart rate, blood pressure, cortisol) variables. Anxiety disorders were assessed at about age 19 years through the Composite International Diagnostic Interview (CIDI). Univariate and multivariate logistic regression analyses were performed with onset of anxiety disorder as a dependent variable and the above-mentioned putative risk factors as predictors. Of the total sample, 25.7% had a lifetime diagnosis of anxiety disorder at age 19 years. Anxiety disorders were twice as prevalent in girls as in boys. Multivariate logistic regression analysis showed that being female (OR = 2.38, *p* < .01), parental depression and anxiety (OR = 1.34, *p* = .04), temperamental frustration (OR = 1.31, *p* = .02) and low effortful control (OR = 0.76, *p* = .01) independently predicted anxiety disorders. We found no associations between biological factors and anxiety disorder. After exclusion of adolescents with an onset of anxiety disorder before age 12 years, being female was the only significant predictor of anxiety disorder. Being female was the strongest predictor for the onset of anxiety disorder. Psychological and parental psychopathology factors increased the risk of diagnosis of anxiety, but to a lesser extent. Biological factors (heart rate, blood pressure, cortisol, and BMI), at least as measured in the present study, are unlikely to be useful tools for anxiety prevention and intervention strategies.

**Electronic supplementary material:**

The online version of this article (10.1007/s00787-020-01669-3) contains supplementary material, which is available to authorized users.

## Introduction

Anxiety disorders are the most common group of mental disorders worldwide [1], and the sixth leading cause of disability worldwide according to the Global Burden of Disease Study [2]. The majority of anxiety disorders have their onset between early adolescence and young adulthood [3–6]. Most adolescents with anxiety disorders do not receive mental health treatments for their symptoms [7]. This is a reason for concern, because untreated adolescent anxiety disorders tend to persist for a long time, with severe consequences [8–10]. Therefore, improving early anxiety prevention and intervention can save substantial dysfunction and suffering [11].

Adolescent anxiety disorders have multifactorial causes, and tend to cluster in specific subgroups. They are more likely to occur in girls than boys [12–15], and have been associated with low family socioeconomic status (SES) [16, 17] and with parental internalizing problems [17–20]. The familial nature of anxiety disorders is assumed to be partly genetic [21] and partly due to social factors. Parents with anxiety and depression may have limited social resources and, as a result, a reduced capacity to help their offspring cope with stressful social situations [22], which may in turn increase the risk of anxiety disorders. In addition to parental internalizing problems, anxiety disorders have been linked to early adverse experiences such as loss of parents, parental divorce, physical and sexual abuse [23–25]. Stressful life events, especially when experienced in childhood, can have long lasting effects in certain regions of the brain that change its developmental trajectory [26] and may lead to the development of psychiatric disorders, including anxiety [27].

Research on the role of child temperament has consistently found that behavioral inhibition (shyness) predicts later anxiety disorder [28–30]. Less studies focused on associations between other temperament dimensions, such as frustration and effortful control, and anxiety disorders. Analyses in the same sample as used in the present study indicated that high frustration is associated with both internalizing and externalizing problems, even when these dimensions are adjusted for each other. Low effortful control was also associated with internalizing problems in these studies, but mainly through its association with externalizing problems [31, 32]. Frustration and effortful control were also related to anxiety in particular [33]. Two interrelated components of effortful control are attentional control, which refers to the capacity to focus and organize attention, and inhibition control, which refers to the ability to control conscious thought and to inhibit or delay a prepotent response [34]. Whereas low inhibition control has been hypothesized to predispose children to externalizing problems, low attention control has been proposed as a risk factor for internalizing problems including anxiety [35].

Whereas psychosocial risk factors have been identified in many cross-sectional and longitudinal studies, relatively few studies have investigated associations between biological factors and anxiety disorders in adolescents. A potentially relevant physiological factor is a dysfunctioning hypothalamic–pituitary–adrenal (HPA) axis. Exposure to stress activates the HPA axis, which results in the secretion of cortisol by the adrenal cortex. Prolonged secretion of cortisol in response to repeated or chronic stressors may up- or down-regulate the HPA axis [36]. Both excessive and insufficient activation of HPA axis have been associated with the development of anxiety disorders [37]. Cortisol levels have been linked with anxiety disorder among child and adolescents both cross-sectionally [38] and longitudinally [39]. Another biological factor of interest is the autonomic nervous system (ANS), which consists of a sympathetic and a parasympathetic branch. In general, the sympathetic system stimulates and the parasympathetic system inhibits bodily responses to stress. The ANS controls cardiovascular responses in particular. Low parasympathetic (vagal) reactivity [40] and a low threshold for sympathetic activation [41] have been proposed as mechanisms underlying the development of anxiety. Children or adolescents with an anxiety disorder had a higher heart rate and systolic blood pressure than those without an anxiety disorder in observational as well as experimental studies [42, 43]. In previous studies on the same general population sample of adolescents as used in the present study, we found cross-sectional associations of heart rate with internalizing symptoms [44], but not with anxiety symptoms [45], and heart rate did not predict anxiety symptoms 2 years later [46]. We are not aware of any other longitudinal studies on associations between autonomic nervous system measures and anxiety disorders in adolescents. Early childhood adversities can lead to hypothalamic pituitary adrenal (HPA) dysfunction and changes in ANS (parasympathetic and sympathetic) responses to stress. Dysregulated stress systems may reflect ineffective stress coping strategies. A consistently low vagal tone has been associated with a reduced adaptational ability in behavioral and cognitive functioning, which in turn increases the probability of developing an anxiety disorder [46, 47]. Furthermore, a low threshold for HPA axis activation has been proposed to reflect sensitivity to adversities, which can lead to anxiety problems over time as well [48].

Obesity, a potential risk factor for anxiety at the cross-roads of biology and psychology, has gained importance because of young people’s increasing body mass index over the past decades [49]. Obesity has obvious biological consequences, for instance through its influence on the HPA axis [50], but also a non-negligible psychosocial component. Adolescents often care about their body image, appearance, and their weight. Higher body mass index (BMI) is associated with greater body image dissatisfaction and negative body weight perception [51]. This could lead to low self-esteem and to social withdrawal or social anxiety, and so increase the probability to develop anxiety disorder among adolescents [52]. Indeed, obese girls have been reported to have almost four times increased risk of developing anxiety disorder compared to normal-weight adolescents [53]. In addition, obesity has been positively associated with panic attacks, social phobia and other anxiety disorders [54]. The association between obesity and anxiety may also be mediated biologically. Chronically increased glucocorticoid levels can promote adipose tissue depots, preferentially within the abdomen, and so cause overweight. In turn, being overweight can disrupt glucocorticoid secretion and maintain high glucocorticoid exposure [55]. Increased glucocorticoid exposure has been implicated not only in the etiology of obesity, but in that of anxiety disorders as well [56]. Overweight can lead to increases in glucocorticoid exposure [57], which can in turn promote further increases in adipose tissue as well as increase the risk of anxiety disorders [48, 58]. Moreover, obesity can lead to increases in inflammatory cytokines [59]. Circulating inflammatory cytokines reach the brain at the level of the hippocampus and amygdala and initiate local inflammation [60], which may lead to anxiety disorders [61].The above-mentioned studies examined whether a specific risk factor was related to anxiety symptoms and disorders, but did not examine psychosocial and biological risk factors of onset of anxiety disorders during adolescence simultaneously. Investigating all factors simultaneously allows for a more complete understanding of the main risk factors of anxiety disorder, because it can show which factors are independent predictors of anxiety disorder onset. Recent studies have highlighted that the extent to which risk factors associated with adolescent’s anxiety disorders are independent factors has remained unclear thus far [62, 63].

The diagnostic class of anxiety disorders consists of a heterogeneous group of disorders [3, 62], among which are separation anxiety disorder, social anxiety disorder, panic disorder, specific phobia, and generalized anxiety disorder, to name a few [64]. These anxiety disorders may be differentially related to the risk factors described above, but this possibility has rarely been investigated in a single cohort. Prior studies suggest that, whereas several factors (e.g., female sex [14, 17, 65–67], a parental history of mental disorders [20, 68, 69], low SES [5, 6]) increases the risk for multiple anxiety disorders, shyness has mostly been related to the development of SAD [28–30, 70] and may be a more specific risk factor. With regard to childhood adversity, the existing evidence does not clearly indicate relationships with specific anxiety disorders in particular, but the findings are mixed [68, 71, 72] and preclude strong conclusions. Relatively little is known on whether biological predictors predict the onset of specific anxiety disorders differentially, but social anxiety disorder has been associated with a high cortisol awaking response [39, 73] and specific phobia with obesity [74].

We investigated a wide range of factors for the onset of anxiety disorders using data of the TRacking Adolescents’ Individual Lives Survey (TRAILS). TRAILS is a Dutch prospective cohort study, which has followed the development of mental and physical health from early adolescence up into adulthood. The primary aim of the current study was to analyze the association of socio-demographic factors, parental psychopathology, childhood adversity, child temperament, heart rate, blood pressure, cortisol, and BMI with the onset of an anxiety disorder during childhood or adolescence. The secondary aim was to investigate whether predictors differed for separate anxiety disorders.

## Methods

### Participants

We studied data from the first and fourth assessment wave of the longitudinal study TRAILS. TRAILS aims to contribute to the understanding of various determinants of mental and physical health by following Dutch pre-adolescents from about 11 years of age onward up into adulthood. The target sample consisted of preadolescents from 135 schools in five municipalities in the North of the Netherlands, including both urban and rural areas. The sampling procedure and response rates of TRAILS have been described in more detail elsewhere [75]. Of all individuals who were approached (*n *= 3145), 210 (6.7%) were excluded because of severe mental retardation, a severe physical illness, or language limitations. Of the remaining 2935 preadolescents, 76% participated in the study (*n* = 2230, mean age 11.09 years, SD 0.55, 51% female). The first assessment wave took place in 2001–2002. Of the 2230 baseline participants, over 84% (*n* = 1881, mean age 19.1, SD = 0.6, 52% girls) were involved in the fourth wave of the study (T4), about 7–8 years later in 2008–2010. Of all T4 participants, 1584 (71% of baseline sample) agreed to have a diagnostic interview (mean age 19.1 years, SD = 0.6, 52% female). As shown in Table S3, compared to adolescents who participated up until the fourth assessment waves, those who dropped out were comparable with regard to parental depression and anxiety and cortisol levels, but more likely to be male, to come from low- and middle-SES families, to report high effortful control, and to have low BP or high BMI at baseline. Each study wave was approved by the Dutch Central Committee on Research Involving Human Subjects (CCMO) and conducted according to the principles of the Declaration of Helsinki.

## Measures

### Anxiety disorders

At the fourth assessment wave, we used the World Health Organization Composite International Diagnostic Interview (CIDI), version 3.0 to assess anxiety disorder according to the Diagnostic and Statistical Manual of Mental Disorders (DSM-IV). The CIDI is a structured diagnostic interview, which has been used in a large number of studies and has shown to have a good reliability and validity [76, 77]. In addition to the lifetime prevalence of psychiatric disorders, the CIDI also obtains age of onset. Anxiety disorders assessed by the CIDI include agoraphobia, generalized anxiety disorder, panic disorder, separation anxiety disorder, social phobia, and specific phobia. The presence of at least one of these disorders was labeled as the presence of an anxiety disorder. In case of multiple anxiety disorders, the first age of onset was taken as age of onset.

### Risk factors

Potential risk factors were assessed at the baseline measurement (age 10–12) and included socio-demographic (sex, socioeconomic status), familial (parental anxiety and depression), psychological (child adversity and temperament), and biological (cortisol, heart rate, blood pressures, and body mass index) variables.

*Socioeconomic status (SES)* was calculated as the average of five standardized variables: family income, educational level (father and mother), and occupational level (father and mother), using the International Standard Classification of Occupations [78]. The lowest 25%, intermediate 50% and highest 25% of the scores were considered to represent low, intermediate and high SES, respectively [79].

*Paternal and maternal anxiety and depression* was measured with the TRAILS Family History Interview [32], for both parents, based on a single informant, typically the mother. Questions were: “Did the mother/father ever have depressive complaints?” and “Did the mother/father ever have anxiety complaints?” Parental anxiety and depression were considered present if at least one of the parents had had depressive or anxiety complaints. Each question was introduced by a vignette describing the main DSM-IV characteristics of the disorders (available on request). The prevalence rates in mother and fathers were, respectively, 27% and 15% for depression, and 16% and 6% for anxiety; and largely comparable to lifetime rates based on diagnostic interviews [80], except for fathers’ rate of anxiety disorder, which was relatively low (for more details see [32]).

*Temperament* was assessed by the Early Adolescent Temperament Questionnaire-Revised (EATQ-R) [81]. We used the parent version because its factor structure was superior to that of the child version in our sample [31]. We included four temperament factors: Shyness (behavioral inhibition to novelty and challenge, especially social situations; 4 items, α = 0.84), Fearfulness (worrying and unpleasant affect related to the anticipation of distress; 5 items, α = 0.63); Frustration (negative affect related to interruption of ongoing tasks or goal blocking; 5 items, α = 0.74), and Effortful control (the capacity to voluntarily regulate behavior and attention; 11 items, α = 0.86). Each item could be rated on a five-point scale ranging from 1 = hardly ever true to 5 = almost always true. The scale score represent mean item scores. The correlations between the four temperament dimensions ranged between  – 0.02 and  – 0.42 (Table S2). In addition to the full effortful control scale, we constructed a subscale consisting of only items measuring attentional control (the capacity to focus attention as well as to shift attention when desired; 5 items, α = 0.77).

*Childhood adversities* were assessed as part of an interview with one of the parents [25], which included a section on major life events. If the child had experienced the death of a family member or other beloved one, parental divorce, or a long absence from home (> 3 months), childhood adversities were considered present.

*Heart rate (HR)* was measured individually in a quiet room at school. All test assistants were trained extensively, and the measures were completed according to a standardized protocol, with a three-lead electrocardiogram. Children were encouraged to relax and were asked not to move or speak during data acquisition. HR signals (beats per minute [bpm]) were registered for 2 min in a standing position. Both standing and supine HR have been associated with anxiety in prior research [82]; therefore, it is relative to anxiety. To allow exploration of the effects of both high and low HR, the sample was categorized into tertiles: low HR (HR ≤ 88.22 bmp, *n* = 367), intermediate HR (88.22 < HR ≤ 99.36 bmp, *n* = 388), and high HR (HR > 99.36 bmp, *n* = 391).

*Blood pressure (BP)* was measured during the occasion as the HR, by means of a cuff that was fixed around the middle phalanx third finger on the right hand. Spontaneous fluctuations in beat-to-beat BP were recorded continuously using a Portapres device (for more details please see Dietrich et al., 2006) [83] in standing position. Based on their systolic blood pressure (SBP), the participants were categorized into tertiles: low BP (SBP ≤ 95.38 mmHg, *n* = 400), intermediate BP (95.38 < SBP ≤ 112.40 mmHg, *n* = 362) and high BP (SBP > 112.40 mmHg, *n* = 384).

*Cortisol.* Salivary cortisol was collected by means of salivettes. Participants were instructed to collect saliva at two time points during the morning; directly after waking up, while still lying in bed (Cort 1), and half an hour later (Cort 2). The saliva samples were stored at -20ºC until analysis. Competitive solid phase time-resolved fluorescence immunoassays with fluorometric end point detection (DELFIA) were used to determine cortisol concentrations in the saliva samples (for more details see Greaves-Lord et al. 2007) [48]. The mean of both samples was used as a measure of morning cortisol levels. Again, we constructed into tertiles: low cortisol (cortisol ≤ 11.26 nmol/l, *n* = 404), intermediate cortisol (11.26 < cortisol ≤ 15.13 nmol/l, *n* = 430), and high cortisol (cortisol > 15.13 nmol/l, *n* = 413).

*Body mass index (BMI).* Participants’ height and weight were assessed individually at school. BMI, a standard index of a person’s weight in relation to height, was determined for each subject by dividing the weight (kg) by the square of the height (m^2^). Children were divided into three groups as tertiles: low BMI (BMI ≤ 16.35, *n* = 521), intermediate BMI (BMI < 16.35 ≤ 18.59, *n* = 532) and high BMI (BMI > 18.59, *n* = 490). Based on standard cutoff scores for normal weight (< 25 kg/m2), overweight (252–29 kg/m^2^), and obese (30 kg/m^2^) [84], the vast majority of these adolescents (84%) had a normal weight, (12.6%) were overweight, and (2.9%) met the criteria for obesity.

### Statistical analysis

Data were analyzed using the Statistical Package for the Social Sciences (SPSS) version 25. First, we calculated descriptive statistics for each of the predictor variables, for participants with and without a lifetime diagnosis of anxiety disorder, and estimated bivariate associations between each of the predictor variables and anxiety disorder, using logistic regression analyses. These analyses were conducted with and without adjusting for sex. To test of the effects of both high and low HR, BP, cortisol and BMI, these variables were entered as dummy variable with the Intermediate group as reference category. All biological variables (HR, BP, cortisol, BMI) were categorized into tertiles. Tertiles offer the advantage of resulting into equal group sizes and hence equal and maximal power across variables. The intermediate category was chosen as the reference group, because we wanted to investigate the effect of both higher and lower than average values. To explore the influence of these decisions on the results, we performed sensitivity analyses in which the biological measures were included as continuous variables in the regression models. After that, variables that were significantly (*p* < 0.05) associated with anxiety in the sex-adjusted bivariate analyses were entered simultaneously in a multivariate logistic regression model to examine which risk variables were independently related to anxiety disorder. These analyses were repeated for specific anxiety disorders that were present in at least 130 (10 per predictor) participants separately [85]. Afterward, as a sensitivity analysis, we excluded adolescents with an anxiety disorder onset before the age of 12 years (*n* = 268), to preclude the possibility of reverse causality. To explore whether the associations between risk factors and anxiety disorder were similar for boys and girls, we tested interaction effects. If the interaction effect was significant (*p* < 0.05), we presented results for boys and girls separately. We did not test sex differences with regard to the individual anxiety disorders, because we had insufficient power to do so. Finally, as sensitivity analyses, we compared the effects of attentional control with those of effortful control as a whole, and excluded adolescents with an anxiety disorder onset before the age of 12 years.

## Results

Descriptive statistics of all predictor variables are presented in Table 1. Please note that the number of participants with valid data varies somewhat across predictors (parental depression and anxiety *n* = 1363; temperament, heart rate, and blood pressure *n* = 1446; cortisol *n* = 1247). Of all participants, 407 (25.7%) had a lifetime diagnosis of any anxiety disorder; 198 (12.5%) had a social phobia, 183 (11.6%) a specific phobia, 64 (4%) a generalized anxiety disorder, 47 (3%) a separation anxiety disorder, 25 (1.6%) a panic disorder, and 15 (0.9%) agoraphobia. Anxiety disorders were about twice as prevalent in girls as in boys.

**Table 1:** Descriptive statistic of risk factors and anxiety disorder among participants

Predictors	Total (*n* = 1584)	No anxiety disorder (*n *= 1177)	Anxiety disorder (*n* = 407)
Age at baseline, mean (SD)	11.08 (0.56)	11.08 (0.55)	11.09 (0.55)
Sex, *n* (%)			
Female	856 (54.0%)	583 (49.5%)	273 (67.1%)
Male	728 (46.0%)	594 (50.5%)	134 (32.9%)
Socioeconomic status, *n* (%)			
Low	306 (19.6%)	224 (19.2%)	82 (20.8%)
Middle	783 (50.1%)	580 (49.6%)	203 (51.4%)
High	475 (30.3%)	365 (31.2%)	110 (27.8%)
Parental depression and anxiety, *n* (%)			
Yes	476 (34.9%)	336 (32.6%)	140 (42%)
No	887 (65.1%)	694 (67.4%)	193 (58%)
Child temperament, mean (SD)			
EATQ-R: effortful control	3.26 (0.68)	3.29 (0.68)	3.17 (0.69)
EATQ-R: shyness	2.50 (0.88)	2.46 (0.85)	2.58 (0.93)
EATQ-R: Fearfulness	2.40 (0.72)	2.35 (0.71)	2.53 (0.72)
EATQ-R: Frustration	2.77 (0.65)	2.73 (0.64)	2.87 (0.64)
Childhood adversity, *n* (%)			
Yes	1072 (68.9%)	798 (68.7%)	274 (69.4%)
No	484 (31.1%)	363 (31.3%)	121 (30.6%)
Heart rate, *n* (%)			
Low	367 (32.0%)	285 (32.8%)	82 (29.5%)
Intermediate	388 (33.9%)	296 (34.1%)	92 (33.1%)
High	391 (34.1%)	287 (33.1%)	104 (37.4%)
Blood pressure, *n* (%)			
Low	400 (34.9%)	312 (35.9%)	88 (31.7%)
Intermediate	362 (31.6%)	270 (31.1%)	92 (33.1%)
High	384 (33.5%)	286 (32.9%)	98 (35.3%)
Cortisol, *n* (%)			
Low	403 (32.4%)	303 (32.7%)	100 (31.3%)
Intermediate	426 (34.2%)	325 (35.1%)	101 (31.7%)
High	416 (33.4%)	298 (32.2%)	118 (37.0%)
BMI, *n* (%)			
Low	521 (33.8%)	395 (34.4%)	126 (31.9%)
Intermediate	532 (34.5%)	405 (35.3%)	127 (32.2%)
High	490 (31.7%)	348 (30.3%)	142 (35.9%)

*EATQ-R* Early Adolescent Temperament Questionnaire-Revised, *BMI* body mass index

The bivariate associations between the putative risk factors and anxiety disorder are presented in Table 2. Being female, parental depression and anxiety, effortful control, shyness, fearfulness, and frustration were all significantly associated with a lifetime diagnosis of anxiety disorder, as assessed 8 years later. Adjusting for sex hardly changed the ORs, but rendered the effect of shyness insignificant. In sensitivity analyses with attentional control instead of effortful control as a whole, its effect was comparable (Table S5).

**Table 2:** Bivariate associations of putative predictors with a lifetime diagnosis of anxiety disorder, with and without adjusting for sex

Predictors	Unadjusted		*p*	Adjusted for sex		*p*
	OR	95% CI		OR	95% CI	
Age	1.04	0.84–1.26	.73	1.06	0.86–1.29	.59
Sex						
Female	2.08	1.64–2.63	< .001		Adjusted	–
Male		Reference				
Socioeconomic status (SES)						
Low	1.21	0.87–1.69	.25	1.18	0.85–1.65	.32
Middle	1.16	0.89–1.51	.27	1.12	0.85–1.46	.43
High		Reference			Reference	
Parental depression and anxiety						
Yes	1.49	1.16–1.93	< .001	1.47	1.14–1.91	< .001
No		Reference			Reference	
Child temperament						
EATQ-R: effortful control	0.77	0.65–0.92	< .001	0.68	0.57–0.82	< .001
EATQ-R: shyness	1.17	1.02–1.34	.02	1.12	0.98–1.23	.10
EATQ-R: fearfulness	1.41	1.19–1.66	< .001	1.35	1.14–1.60	< .001
EATQ-R: frustration	1.40	1.16–1.68	< .001	1.50	1.24–1.80	< .001
Childhood adversity						
Yes	0.72	0.50–1.06	.09	1.01	0.78–1.29	.96
No		Reference			Reference	
Heart rate						
Low	0.92	0.66–1.30	.65	0.95	0.67–1.34	.77
Intermediate		Reference			Reference	
High	1.16	0.84–1.16	.35	1.09	0.79–1.52	.57
Blood pressure						
Low	0.83	0.59–1.15	.27	0.82	0.58–1.14	.24
Intermediate		Reference			Reference	
High	1.01	0.72–1.40	.97	0.98	0.70–1.37	.91
Cortisol						
Low	1.07	0.78–1.46	.68	1.14	0.82–1.57	.43
Intermediate		Reference			Reference	
High	1.28	0.94–1.75	.11	1.25	0.91–1.71	.16
BMI						
Low	1.01	0.77–1.35	.91	1.05	0.79–1.40	.73
Intermediate		Reference			Reference	
High	1.30	0.98–1.72	.06	1.27	0.96–1.69	.09

*EATQ-R* Early Adolescent Temperament Questionnaire-Revised, *BMI* body mass index

Table 3 shows the effects of the five predictors with significant bivariate associations adjusted for each other. In this multivariate model, the effect of sex, parental depression and anxiety, effortful control, and frustration remained approximately similar and significant. The effect of fearfulness decreased and was no longer statistically significant, indicating that fearfulness was not uniquely associated with anxiety. The effect of attentional control was slightly weaker than that of effortful control, but only marginally significant except social anxiety disorder (Table S6). Exclusion of adolescents with an onset of anxiety disorder before the age of 12 years generally did not affect ORs, but most were not significant anymore, except for the effect of being female.

**Table 3:** Multivariate associations with a lifetime diagnosis of anxiety disorder

Predictors	OR (95% CI)		*p*	OR (95% CI) Excluding early onsets (< 12 years)		*p*
Sex (female)	2.38	1.79–3.18	< .001	3.03	1.87–4.91	< .001
Parental depression and anxiety	1.34	1.02–1.76	.04	1.35	0.88–2.07	.16
EATQ-R: effortful control	0.76	0.61–0.94	.01	0.86	0.61–1.23	.40
EATQ-R: fearfulness	1.13	0.93–1.37	.23	1.07	0.79–1.45	.69
EATQ-R: frustration	1.31	1.04–1.65	.02	1.38	0.96–1.99	.08

EATQ-R Early Adolescent Temperament Questionnaire-Revised

Of all specific anxiety disorders, only social anxiety disorder and specific phobia were prevalent enough to allow separate analyses. Both social and specific phobia showed significant bivariate associations with being female, effortful control, and frustration. In addition, social anxiety disorder was associated with shyness, and specific phobia with high BMI (Table S1). In the multivariate analyses, sex and shyness remained significant predictors of social anxiety disorder (Table 4), while sex and effortful control significantly predicted specific phobia disorder (Table 5). The effects of attentional control were similar to those of effortful control (Table S7). When excluding anxiety disorder at baseline, ORs were comparable. After exclusion of the early onsets, the only predictor that was still significant—and even stronger than before—was being female.

**Table 4:** Multivariate associations with a lifetime diagnosis of Social Anxiety Disorder

Predictors	OR (95% CI)	*p*	OR (95% CI) Excluding early onsets (< 12 years)	*p*
Sex (female)	1.53 (1.09–2.13)	.01	2.35 (1.32–4.22)	< .01
EATQ-R: effortful control	0.84 (0.65–1.09)	.19	0.87 (0.57–1.34)	.54
EATQ-R: shyness	1.51 (1.27–1.81)	< .01	1.26 (0.94–1.70)	.13
EATQ-R: frustration	1.24 (0.95–1.62)	.11	1.31 (0.84–2.04)	.23

*EATQ-R* Early Adolescent Temperament Questionnaire-Revised

**Table 5:** Multivariate associations with a lifetime diagnosis of Specific Phobia

Predictors	OR (95% CI)	*p*	OR (95% CI) Excluding early onsets (< 12 years)	*p*
Sex (female)	2.98 (2.02–4.39)	< .001	3.94 (1.43–10.85)	< .01
EATQ-R: effortful control	0.69 (0.53–0.91)	.01	0.83 (0.42–1.62)	.58
EATQ-R: frustration	1.31 (0.98–1.74)	.06	1.26 (0.63–2.53)	.52
BMI low	0.95 (0.62–1.46)	.81	1.20 (0.46–3.15)	.72
Intermediate	Reference		Reference	
High	1.28 (0.85–1.92)	.23	0.99 (0.35–2.78)	.99

EATQ-R Early Adolescent Temperament Questionnaire-Revised, *BMI* body mass index

Significant sex differences were only found for SES (*p* = 0.023) and HR (*p* = 0.006). With regard to SES, the largest sex differences were found for the middle group: whereas girls in the middle-SES group were more likely to develop an anxiety disorder than girls in the high-SES reference category (OR 1.42, *p* = 0.048), this was not the case for boys in the middle-SES group (OR = 0.74, *p* = 0.18). For low-SES girls and boys, the effects were approximately equal (OR 1.22, *p* = 0.40 and OR 1.18, *p* = 0.53, respectively). With regard to HR, the largest sex differences were found for the high group: girls with a high HR (as compared to the intermediate group) tended to have a lower probability of anxiety (OR 0.82, *p* = 0.32); high-HR boys had a higher probability (OR 2.20, *p* = 0.008). Interestingly, girls and boys with a low HR showed a similar trend, but less pronounced (girls: OR 0.90, *p* = 0.61; boys: OR 1.14, *p* = 0.68).

In a sensitivity analysis with SES included as a continuous variable, its effect was marginally significant (unadjusted: OR 0.85, *p* = 0.04; adjusted for sex OR 0.86, *p* = 0.05), suggesting that the probability of anxiety disorder decreased slightly with increasing parental SES. In addition, sensitivity analysis with HR, BP, cortisol and BMI included as continuous variables yielded similar findings as the original models, that is, none of these variables was significantly associated with anxiety disorder (Table S4).

## Discussion

In this study, we investigated independent risk factors in pre-adolescence (11 years) for anxiety disorders in late adolescence (19 years) using a wide range of factors and a large community sample. In addition, we explored whether predictors differ for various anxiety disorders. In the multivariate analysis, female sex was the strongest predictor of anxiety, followed by parental history of depression and anxiety, temperamental frustration and low effortful control. After exclusion of adolescents with an anxiety disorder at baseline, the only statistically significant factor was being female; the effect estimates of parental depression and anxiety, frustration, and effortful control were comparable in strength but no longer statistically significant. None of the included biological factors predicted the onset of an anxiety disorder. Subtype-specific analyses revealed that being female and shyness were associated with social anxiety disorder, while specific phobia was predicted by female sex and low effortful control. Again, after exclusion of adolescents with an age of onset before 12 years old, only sex remained a statistically significant predictor of the outcome.

Consistent with prior research, we found that being female is an independent and robust risk factor for the development of any anxiety disorder during adolescence. Sex is not a causal risk factor of anxiety disorders [15]; rather, it is a marker of factors and processes that are assumed to be more proximally related to anxiety disorders. The potential mechanisms underlying sex differences in anxiety may occur at two levels. The first level concerns consequences of being male and female that are related to differential prenatal and sex hormone effects on the programming brain [86]. Puberty is characterized by an increase in gonadal steroid hormone secretion (estradiol and testosterone). In girls, this pubertal process of gonadarche starts 1–2 years earlier than in boys and involves a four to nine times increase in estradiol levels [87]. Moreover, the decline in estradiol levels at the end of the luteal phase of the menstrual cycle may increase anxiety symptoms in girls who are sensitive to hormonal fluctuations [88, 89]. Large estradiol fluctuations may enhance activation of the HPA axis, leading to stronger cortisol stress responses and, through that, to increased fear conditioning and so put girls at an elevated risk of developing anxiety disorders [88]. Several other brain regions may be involved in the sex difference in anxiety sensitivity as well, particularly the amygdala and hippocampus. These brain regions are known to be related to stress reactivity and anxiety [61]. During adolescence, the amygdala volume increases significantly more in boys than in girls, whereas hippocampal volume increases faster in girls [90]. This sex difference in volume is augmented by greater densities of testosterone receptors in the amygdale and more estrogen receptors in the hippocampus [90, 91]. Testosterone levels can inhibit HPA axis response to stress and have been found to have anxiolytic effects [92]. Hence, while puberty-related hormonal changes increase girls’ risk of anxiety, these developments tend to be protective for boys. The second level involves gender differences in socio-culturally determined role behaviors that may affect the development of anxiety. Even though traditional role patterns and expectations have diminished considerably over the past decades, men are still expected to be stronger, braver, and more autonomous than women, and the expression of emotions, dependence and vulnerability is more acceptable for women than for men. This may make it easier for girls to talk about their anxiety symptoms than for boys [93], and easier to seek help [94]. Furthermore, compared to men, women are more conditioned to care for others and to engage in close relationships, which makes them more sensitive to interpersonal and psychosocial stress [15, 95]. Stress exposure levels may differ as well, because women are more likely to be the victims of verbal harassment and sexual abuse [96]. Considering the multitude of mechanisms at both the sex and the gender level, it is hardly surprising that girls are at an increased risk to develop an anxiety disorder.

In addition to sex, parental depression and anxiety predicted anxiety disorder, but the effects were weaker than those found in previous longitudinal population-based studies. Whereas the OR for parental depression and anxiety was 1.3 in our study, Hyland et al. [13] reported ORs ranging from 1.5 to 2.4 for the prediction of an anxiety disorder between the ages of 10 and 21 years, Wittchen et al. [17] an OR of 2.6 with regard to the lifetime prevalence of social phobia at age 24 years, and Schreier et al. [19] ORs ranging from 5.0 to 6.3 for the lifetime prevalence of anxiety disorders at age 14–17 years. There are at least three possible reasons for the relatively weak effect estimates of parental depression and anxiety in the current study. First, we studied parental depression and anxiety symptoms at subclinical levels, while Hyland et al. and Schreier et al. assessed parental depression and anxiety based on clinical diagnoses of mood and anxiety disorders. It is quite likely that severe parental anxiety and depression is more strongly associated with offspring anxiety disorders than subclinical anxiety. Second, we interviewed one parent to assess psychopathology in both the mother and father, and combined this information into one variable. This may have introduced excess measurement error and, as a result, the association between parental psychopathology and offspring anxiety disorder may have been underestimated. Third, parental psychopathology may increase the likelihood of offspring anxiety disorder through low SES [17, 97]. Our sample contained relatively few participants living in low-SES families, even within the subgroup with parental anxiety and depression, which may have reduced the strength of the association.

In the current study, SES was not a significant risk factor for anxiety disorder. However, a sensitivity analysis including SES as a continuous variable suggests that higher SES was related to a somewhat reduced risk of anxiety disorder, especially in girls.

Parent-reported shyness was not associated with the aggregated measure of anxiety disorder but did predict social anxiety disorder, as also reported by Essex et al. [29], Hayward et al. [30] and Chronis-Tuscano et al. [28]. That shyness did not predict anxiety in general confirms the findings of a previous TRAILS study based on continuous outcome measures, which found that shyness did not predict general anxiety symptoms at age 17–19 years [33]. It is also consistent with a cross-sectional study by Gladstone et al. [98], in which childhood shyness was associated with adult social anxiety disorder, but not with GAD, panic disorder and agoraphobia. In contrast, parent-reported shyness at age 4 years has been shown to predict childhood SAD, separation anxiety, GAD and specific phobia at age 6 [99]. Perhaps, childhood shyness is a general risk factor of anxiety in young childhood, but a specific predictor of SAD in older age groups. In addition, the positive association between frustration and anxiety and the negative association between effortful control and anxiety have both been found before, also in the same cohort [33]. Our study adds to these previous findings that our outcome measures involved DSM-IV anxiety disorders instead of symptom scores, and we had a longer follow-up period. We found low effortful control to be a risk factor for specific phobia in particular. Including only attentional control items yielded comparable findings. Low attentional control may be particularly relevant to specific phobia [100, 101], because the ability to distract one´s attention from catastrophic and feared conditions may prevent maladaptive thinking patterns and the development of fear [102].

None of the included biological factors (heart rate, blood pressure, cortisol and BMI at age 11) predicted anxiety disorders at age 19 years. Previous studies based on the same sample did point to some links between biological factors and anxiety symptoms: Dietrich et al. [44] found that adolescents with current internalizing problems, including anxiety, had higher HR in supine posture. Greaves-Lord et al. [45, 46], on the other hand, did not find evidence for associations between anxiety symptoms and HR, neither cross-sectionally [45] nor prospectively [46]. Both Greaves-Lord et al. [48] and Dietrich et al. [103] found cross-sectional associations between high cortisol levels and anxiety, but no prospective associations [104]. A few other studies found that higher HR and BP were cross-sectionally associated with anxiety symptoms [41, 82] and anxiety disorder [42, 105], but these mostly included small, nonrepresentative samples, limiting the generalizability of the findings. We are not aware of any prospective studies on the association between HR and anxiety disorders or BP and anxiety disorders among adolescents. We found one prospective study with regard to cortisol: Adam et al. [39] found that a high cortisol awaking response at age 16 strongly predicted a combined measure of anxiety disorder and social anxiety disorder at age 23. These diverging results may be due to methodological differences. Whereas Adam et al. [39] assessed the CAR during three consecutive days, we only used during a single day; hence, the reliability of our cortisol measurement is probably inferior to theirs. Yet the lack of effects in our study calls for restraint when drawing conclusions about the role of biological factors such as HR and cortisol, at least as measured in this study, in the etiology of anxiety disorders. It seems premature to presume these factors play an important role.

We found that high HR predicted anxiety disorder in boys, whereas no significant association was found in girls. It should be noted that Greaves-Lord et al. [45, 46], who used the same sample but continuous anxiety measures and a shorter follow-up period, found no evidence for an association between HR and anxiety symptoms in boys (or girls), cross-sectionally [48] or prospectively [46]. Rogeness et.al [43] did find that boys with high HR in adolescence were at increased risk for anxiety disorder. Whereas boys generally show stronger physiological stress responses than girls, girls tend to respond with more intense reported anxiety [106, 107]. Higher HR might therefore reflect unexpressed (and perhaps even subconscious) anxiety that increases the risk of future anxiety disorder in boys.

As opposed to findings reported by Anderson et al. [53], high BMI did not predict later anxiety disorder in our study. Anderson et al. used parent-reported height and weight in their study, instead of objective measurements like we did. Over- or underestimation may have influenced their findings, but is an unlikely explanation for the difference in effects. Possibly, this difference is due to the greater number of obese adolescents in Andersonʹs sample, as compared to ours.

Our study was conducted with the hope that its results could be used to improve prevention and early intervention programs for anxiety disorders, by providing guidance regarding which individuals to target. In that respect, the finding that sex was the only predictor that survived adjustment for other variables and for pre-assessment anxiety was somewhat disappointing. Numerous studies have suggested that female sex is an important marker of anxiety sensitivity before; among other things, it has been associated with a higher severity of symptoms, more chronic and recurrent anxiety disorder, and greater homotypic and heterotypic continuity [108, 109]. Nevertheless, not much is known about the role of sex and gender differences in treatment outcomes of and barriers to seek for anxiety disorders.

Our study has a number of strengths. It is based on a large community sample that was followed for over 8 years, which allowed us to examine a wide range of potential risk factors in pre-adolescence (11 years) and how these relate to onset of anxiety disorder in the period from pre-adolescence to young adulthood (19 years). Previous studies mainly focused on anxiety symptoms in adolescence, while we focused on the development of a clinical diagnosis of anxiety disorder in adolescents, which is another strength of our study.

There are several limitations as well. First, the power to detect effects was relatively limited, particularly for some of the specific anxiety disorders. To be more specific, we could not estimate the effects of the risk factors for agoraphobia, separation anxiety, panic disorder, and GAD, because the low relatively prevalence’s of these disorders implied fewer than 10–15 observations per predictor. Furthermore, in a sensitivity analysis we excluded adolescents with an early (i.e., before the age of 12 years) onset of anxiety. Several associations lost statistical significance, although the effect sizes were of comparable strength, suggesting that the resulting sample size may have been too small to detect these effects. Second, for the biological factors, participants were divided into three about equally sized groups. We used this categorization to have adequate group sizes, but it should be noted that we did not use cutoffs according to standardized guidelines. Third, in our sensitivity analysis we used retrospectively gathered information on age of onset of anxiety disorders, which might have led to inaccurate representations. Fourth, anxiety disorders were assessed once, using an interview that assessed lifetime presence of anxiety disorder. This may have introduced recall failure and hence underestimations of the lifetime prevalence rates of the anxiety disorders [110]. Although recall bias probably plays a lesser role in adolescent than in adult samples, we cannot exclude that we have missed cases, and that this misclassification-biased associations between risk factors and anxiety disorder [111]. Fifth, we did not include stress reactivity measures. Laboratory studies using psychosocial stress tests found that stress-related changes in heart rate, blood pressure, and cortisol were more strongly associated with anxiety disorder than resting states [112, 113], hence our findings do not preclude that physiological stress reactivity measures predict future anxiety disorders. Sixth, we measured putative risk factors during pre-adolescence, but anxiety levels may already have been increased before that. Risk factors assessed during early childhood might be more robust predictors, and more valuable targets for early prevention and intervention [114]. Seventh, when analyzing the associations between risk factors and anxiety disorders we did not adjust for the presence of other psychiatric disorders, and therefore the associations may not be specific for anxiety disorders. Yet, since anxiety disorders generally have an earlier age of onset than most other psychiatric disorders, we do not consider it likely that the associations found were merely indirect and mediated by other disorders with an earlier onset.

To conclude, we examined a wide range of factors for the onset of anxiety disorders and showed that only sex was consistently and strongly associated to anxiety disorders in all analyses. Temperament and parental depression and anxiety increased the risk of anxiety disorder to a lesser extent. Biological factors (heart rate, blood pressure, cortisol, and BMI), at least as measured in the present study, are unlikely to be useful tools for anxiety prevention and intervention strategies. Some predictors were anxiety-subtype specific; shyness predicted particularly social anxiety disorder and low effortful control particularly predicted specific phobia.

## Electronic supplementary material

Below is the link to the electronic supplementary material.

Supplementary material 1 (DOCX 28 KB)
