# Supplementary material for: Psychosocial and biological risk factors of anxiety disorders in adolescents: a TRAILS report
Source: Eur Child Adolesc Psychiatry. 2020 Oct 28;30(12):1969–82. doi: 10.1007/s00787-020-01669-3 (PMC8563629; doi:10.1007/s00787-020-01669-3)
Supplement: Supplementary file 1 — Supplementary material 1 (DOCX 28 KB) [file 787_2020_1669_MOESM1_ESM.docx]

**Appendix**

**Table S1.** Bivariate associations of putative predictors with a lifetime diagnosis of Social anxiety disorder and Specific phobia, with and without adjusting for sex.

| Predictors | Social Phobia | | Specific Phobia | |
| --- | --- | --- | --- | --- |
|  | Unadjusted  OR (95% CI) | Adjusted  OR (95% CI) | Unadjusted  OR (95% CI) | Adjusted  OR (95% CI) |
| Age | 0.96 (0.74–1.26) | 0.97 (0.75–1.27) | 0.95 (0.72–1.25) | 0.97 (0.74–1.29) |
| Sex | | | | |
| Female | 1.46 (1.07–1.98)* | Adjusted | 2.42 (1.72–0.39)** | Adjusted |
| Male | Reference |  | Reference |  |
| Socioeconomic status (SES) | | | | |
| Low | 0.80 (0.52–1.23) | 0.78 (0.51–1.21) | 1.55 (0.98–2.45) | 1.51 (0.95–2.40) |
| Middle | 0.80 (0.57–1.11) | 0.78 (0.55–1.09) | 1.46 (0.99–2.14) | 1.39 (0.95–2.05) |
| High | Reference | Reference | Reference | Reference |
| Parental depression and anxiety | | | | |
| Yes | 1.38 (0.99–1.91) | 1.36 (0.98–1.90) | 1.44 (1.02–2.03)* | 1.41(0.99–2.00) |
| No | Reference | Reference | Reference | Reference |
| Child temperament | | | | |
| EATQ-R: effortful control | 0.83 (0.66–1.03) | 0.77 (0.61–0.97)* | 0.71 (0.56–0.90)** | 0.61(0.48–0.78)** |
| EATQ-R: shyness | 1.56 (1.31–1.86)** | 1.53 (1.29–1.84)** | 0.96 (0.79–1.15) | 0.90 (0.75–1.09) |
| EATQ-R: fearfulness | 1.13 (0.91–1.40) | 1.10 (0.89–1.37) | 1.32 (1.05–1.65)* | 1.24 (0.99–1.56) |
| EATQ-R: frustration | 1.35 (1.06–1.72)* | 1.40 (1.09–1.79)** | 1.42 (1.10–1.82)** | 1.54 (1.19–1.99)** |
| Childhood adversity | | | | |
| Yes | 1.05 (.76–1.46) | 1.04 (.75–1.45) | 1.12 (.79–1.57) | 1.07(.77–1.54) |
| No | Reference | Reference | Reference | Reference |
| Heart rate | | | | |
| Low | 1.04 (0.66–1.64) | 1.05 (.66–1.66) | 0.66 (0.41–1.07) | 0.68 (0.42–1.10) |
| High | 1.00 (0.63–1.56) | 0.96 (.61–1.51) | 1.14 (0.75–1.74) | 1.07 (0.69–1.64) |
| Intermediate | Reference | Reference | Reference | Reference |
| Blood pressure | | | | |
| Low | 0.74 (0.47–1.17) | 0.74 (0.47–1.16) | 0.81 (0.52–1.25) | 0.80 (0.51–1.25) |
| High | 0.86 (0.55–1.35) | 0.85 (0.55–1.34) | 0.74 (0.47–1.16) | 0.71 (0.45–1.13) |
| Intermediate | Reference | Reference | Reference | Reference |
| Cortisol | | | | |
| Low | 1.14 (0.76–1.71) | 1.17 (0.78–1.76) | 1.07 (0.68–1.67) | 1.15 (0.73–1.80) |
| High | 0.96 (0.63–1.45) | 0.94 (0.62–1.42) | 1.44 (0.94–2.19) | 1.39 (0.91–2.13) |
| Intermediate | Reference | Reference | Reference | Reference |
| BMI | | | | |
| Low | 1.10 (.75–1.61) | 1.12 (.77–1.64) | 1.07 (.71–1.60) | 1.11 (.74–1.67) |
| High | 1.31 (.91–1.91) | 1.30 (.90–1.89) | 1.51 (1.03–2.22)* | 1.47 (1.00–2.17)* |
| Intermediate | Reference | Reference | Reference | Reference |

*significant at p<.05, ** significant at p<.01. EATQ–R Early Adolescent Temperament Questionnaire–Revised, BMI Body Mass Index, Adjusted OR: adjust for sex.

**Table S2.** Correlations between child temperaments.

|  | EATQ-R: effortful control | EATQ-R: fear | EATQ-R: shyness | EATQ-R: frustration |
| --- | --- | --- | --- | --- |
| EATQ-R: effortful control | 1 | – | – | – |
| EATQ-R: fear | –0.24*** | 1 | – | – |
| EATQ-R: shyness | –0.02 | 0.15*** | 1 | – |
| EATQ-R: frustration | –0.41*** | 0.31*** | 0.10*** | 1 |
| **. Correlation is significant at the 0.01 level (2–tailed). | | | | |

**Table S3.** Bivariate logistic regression analysis predicting T1–to–retainers and T1 to–dropouts. Retention and dropout observed at T4.

| Predictors | T1– retainer  (n=1584) | T1– dropout  (*n*=646) | | |
| --- | --- | --- | --- | --- |
|  | *n* *%* | *n %* | OR | 95% CI |
| Sex, *n* (*%)* | | |  |  |
| Female | 856 (54.0%) | 276 (42.7%) | Reference | |
| Male | 728 (46.0%) | 370 (57.3% | 1.56 | 1.31–1.90 *** |
| Socioeconomic status, *n (%)* | | |  |  |
| Low | 306 (19.6%) | 247 (39.6%) | 5.05 | 3.75–6.77*** |
| Middle | 783 (50.1%) | 301 (48.2% | 2.40 | 1.83–3.17*** |
| High | 475 (30.3%) | 76 (12.2%) | Reference | |
| Parental depression and anxiety, *n (%)* | | |  |  |
| Yes | 476 (34.9%) | 202 (37.9%) | 1.14 | 0 .92–1.40 |
| No | 887 (65.1%) | 331 (62.1%) | Reference | |
| Child temperament, mean (SD) | | |  |  |
| EATQ-R: effortful control  EATQ-R: shyness  EATQ-R: fearfulness  EATQ-R: frustration | 3.26 (0.68)  2.50 (0.88)  2.40 (0.72)  2.77 (0.65) | 3.11 (0.66)  2.48 (0.75)  2.56 (0.91)  2.83 (0.68) | 0.73  1.09  1.09  0.96 | 0.61–0.85*******  0.94–1.26  0.97–1.22  0.81–1.14 |
| Childhood adversity, *n (%)* | | |  |  |
| Yes | 1072 (68.9%) | 453 (72.9%) | 1.21 | 0.98–1.49 |
| No | 484 (31.1%) | 168 (27.1%) | Reference | |
| Heart rate, *n (%)* | | |  |  |
| Low | 367 (32.0%) | 165 (36.6%) | 1.20 | 0.92–1.56 |
| Intermediate | 388 (33.9%) | 145 (32.2%) | Reference | |
| High | 391 (34.1%) | 141 (31.2%) | 0.96 | 0.73–1.26 |
| Blood pressure, *n* *(%)* | | |  |  |
| Low | 400 (34.9%) | 132 (29.3%) | 0.69 | 0.53–0.91** |
| Intermediate | 362 (31.6%) | 171 (37.9%) | Reference | |
| High | 384 (33.5%) | 148 (32.8) | 0.80 | 0.62–1.04 |
| Cortisol, *n* *(%)* | | |  |  |
| Low | 403 (32.4%) | 152 (36.2%) | 1.29 | 0.98–1.70 |
| Intermediate | 426 (34.2%) | 126 (30.0%) | Reference | |
| High | 416 (33.4%) | 142 (33.8%) | 1.18 | 0.89–1.55 |
| BMI, *n* *(%)* | | |  |  |
| Low | 521 (33.8%) | 199 (32.2%) | 1.07 | 0.85–1.36 |
| Intermediate | 532 (34.5%) | 189 (30.6%) | Reference | |
| High | 490 (31.7%) | 230 (37.2%) | 1.32 | 1.05–1.66* |

*p<.05; ** p<.01, ***p<.001, Odds ratios (OR) and 95% confidence intervals (CI), EATQ–R Early Adolescent Temperament Questionnaire–Revised, BMI–Body Mass Index

| **Table S4.** Bivariate associations of continuous biological measures with a lifetime diagnosis of anxiety disorder, with and without adjusting for sex. | | | | | | |
| --- | --- | --- | --- | --- | --- | --- |
| Predictors | Unadjusted | | *p* | Adjusted for sex | | *p* |
|  | OR | 95% CI |  | OR | 95% CI |  |
| Heart rate | 1.09 | 0.95–1.24 | .23 | 1.05 | 0.92–1.21 | .47 |
| Blood pressure | 1.05 | 0.92–1.19 | .49 | 1.04 | 0.91–1.19 | .54 |
| Cortisol | 1.06 | 0.94–1.21 | .35 | 1.02 | 0.90–1.17 | .74 |
| BMI | 1.11 | 0.99–1.25 | .07 | 1.09 | 0.97–1.23 | .16 |
| BMI= Body Mass Index. | | | | | | |

| **Table S5.** Bivariate associations of attention control with a lifetime diagnosis of anxiety disorders, with and without adjusting for sex. | | | | | | | | | | | | |
| --- | --- | --- | --- | --- | --- | --- | --- | --- | --- | --- | --- | --- |
| Predictors | Anxiety disorder | | | | Social anxiety disorder | | | | Specific phobia | | | |
|  | Unadjusted  OR (95% CI) | | Adjusted  OR (95% CI) | | Unadjusted  OR (95% CI) | | Adjusted  OR (95% CI) | | Unadjusted  OR (95% CI) | | Adjusted  OR (95% CI) | |
| EATQ–R: Attention Control | 0.80 | 0.68–0.93** | 0.72 | 0.61–0.85*** | 0.90 | 0.73–1.12 | 0.86 | 0.90–1.07 | 0.72 | 0.58–0.89** | 0.64 | 0.51–0.80*** |
| ** significant at p<.01, ***significant at p<.001, EATQ–R Early Adolescent Temperament Questionnaire–Revised, Adjusted OR: adjust for sex. | | | | | | | | | | | | |

**Table S6.** Multivariate associations with a lifetime diagnosis of anxiety disorder (attention control).

| Predictors | OR (95% CI) | | *p* | OR (95% CI)  Excluding early onsets (< 12 years) | | *p* |
| --- | --- | --- | --- | --- | --- | --- |
| Sex (female) | 2.32 | 1.75–3.09 | <.001 | 3.02 | 1.87–4.87 | <.001 |
| Parental depression and anxiety | 1.35 | 1.03–1.78 | .03 | 1.35 | 0.88–2.08 | .17 |
| **EATQ-R: attention control** | 0.83 | 0.68–1.01 | .06 | 0.86 | 0.62–1.19 | .36 |
| EATQ-R: fearfulness | 1.13 | 0.93–1.37 | .23 | 1.06 | 0.78–1.44 | .72 |
| EATQ-R: frustration | 1.33 | 1.05–1.68 | .02 | 1.37 | 0.94–1.99 | .09 |

EATQ–R Early Adolescent Temperament Questionnaire–Revised

**Table S7.** Multivariate associations with a lifetime diagnosis of Specific Phobia (attention control)

| Predictors | OR (95% CI) | *p* | OR (95% CI)  Excluding early onsets (< 12 years) | *p* |
| --- | --- | --- | --- | --- |
| Sex (female) | 2.93 (1.99–4.31) | <.001 | 3.93 (1.44–10.75) | .01 |
| **EATQ-R: attention control** | 0.72 (0.56–0.93) | .01 | 0.81 (0.43–1.51) | .51 |
| EATQ-R: frustration | 1.29 (0.97–1.73) | .08 | 1.23 (0.61–2.50) | .57 |
| BMI |  |  |  |  |
| Low | 0.95 (0.62–1.47 | .83 | 1.20 (0.46–3.16) | .71 |
| Intermediate | Reference |  | Reference |  |
| High | 1.28 (0.86–1.93) | .23 | 0.99 (0.36–2.79) | .99 |

EATQ–R Early Adolescent Temperament Questionnaire–Revised, BMI–Body Mass Index
